# The Hippo-YAP pathway in various cardiovascular diseases: Focusing on the inflammatory response

**DOI:** 10.3389/fimmu.2022.971416

**Published:** 2022-08-18

**Authors:** Ancheng Zheng, Qishan Chen, Li Zhang

**Affiliations:** Department of Cardiology and Institute for Developmental and Regenerative Cardiovascular Medicine, Xinhua Hospital, School of Medicine, Shanghai Jiaotong University, Shanghai, China

**Keywords:** Hippo-YAP pathway, inflammation, immune, myocardial infarction, atherosclerosis

## Abstract

The Hippo pathway was initially discovered in *Drosophila melanogaster* and mammals as a key regulator of tissue growth both in physiological and pathological states. Numerous studies depict the vital role of the Hippo pathway in cardiovascular development, heart regeneration, organ size and vascular remodeling through the regulation of YAP (yes-associated protein) translocation. Recently, an increasing number of studies have focused on the Hippo-YAP pathway in inflammation and immunology. Although the Hippo-YAP pathway has been revealed to play controversial roles in different contexts and cell types in the cardiovascular system, the mechanisms regulating tissue inflammation and the immune response remain to be clarified. In this review, we summarize findings from the past decade on the function and mechanism of the Hippo-YAP pathway in CVDs (cardiovascular diseases) such as myocardial infarction, cardiomyopathy and atherosclerosis. In particular, we emphasize the role of the Hippo-YAP pathway in regulating inflammatory cell infiltration and inflammatory cytokine activation.

## Introduction

The Hippo-YAP (yes-associated protein) pathway, which was originally identified in the Drosophila genus and is highly conserved in mammals, consists of a kinase cascade as well as downstream effectors and the transcriptional coactivators (1). These core components of the Hippo pathway control transcriptional programs involved in cell growth, development, and organ size. CVDs (cardiovascular diseases) remain the leading cause of death worldwide (2–4). Numerous studies have revealed a pivotal role of the Hippo–YAP pathway and its upstream regulators in cardiac development, growth, homeostasis, disease, and regeneration using genetic models and biochemical studies (5–8).

Multiple lines of evidence continue to support that inflammation is a central process, along with multiple infiltrated immune cells and activated inflammatory cytokines, in the development of various CVDs, such as myocardial infarction (MI) (9–11), cardiomyopathy (12, 13) and atherosclerosis (14, 15). Emerging evidence shows that targeting specific inflammatory proteins or pathways can be an effective method to treat CVDs (16, 17). However, to date, there have been no clinical drugs targeting inflammation to treat CVDs; thus, more targets, pathways and their mechanisms need to be clarified.

Although the biological functions of the Hippo pathway in CVDs have been studied extensively over the last twenty years, the inflammatory regulation of this pathway, particularly in mammals, is poorly understood. Recently, the role of the Hippo-YAP pathway in regulating inflammation and the immune response has attracted considerable attention (18–25). Considering the controversial roles of the Hippo-YAP pathway in different contexts and cell types in the cardiovascular system, summarizing and clarifying the specific modulatory effect of the Hippo-YAP pathway on physiological and pathological cardiovascular system immunity and inflammation is indispensable for further clinical study. In this review, we summarize the progress on the Hippo-YAP pathway in CVDs in recent years, especially in immune and inflammatory areas.

## Introduction of the Hippo-YAP pathway

### Overview of the Hippo-YAP pathway

**Figure 1:**
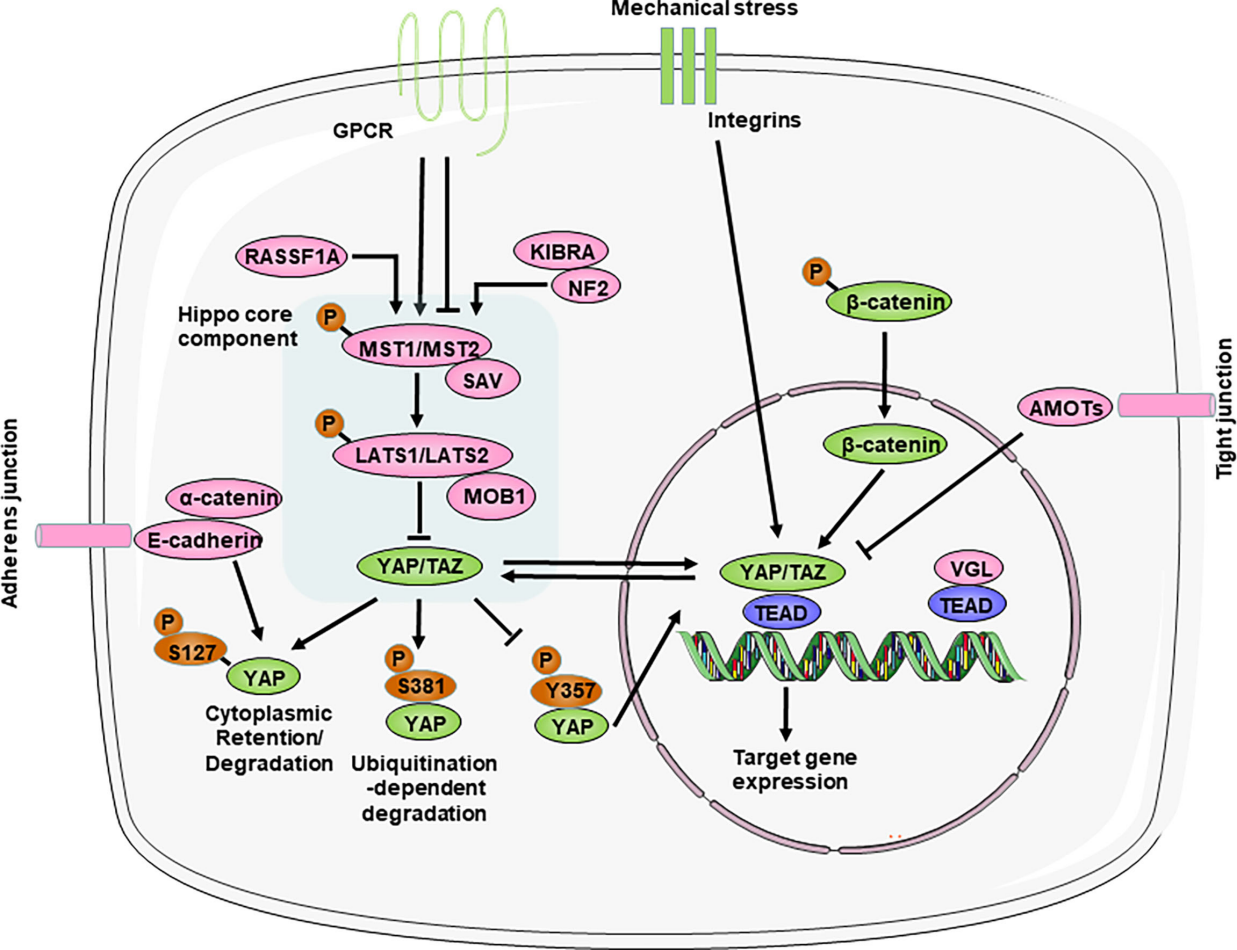
Overview of the Hippo-YAP pathway. The core components of the Hippo-YAP pathway contain kinases mammalian Ste20-like kinases 1/2 (MST1/2) and Large tumor suppressor homolog 1/2 (LATS1/2), their cofactors Salvador (SAV) and scaffolding proteins MOB domain kinase activator 1A/B (MOB1A/B), the transcription co-activators Yes-associated protein (YAP) and transcriptional co-activator with PDZ-binding motif (TAZ) and the TEAD1–TEAD4 family of transcription factors. When the Hippo-YAP pathway is at the “ON” status (red), phosphorylated MST1/2 activates the phosphorylation of LATS1/2, which in turn phosphorylate and promote the degradation of the YAP and TAZ. When the Hippo-YAP pathway in at the “OFF” status (green), YAP/TAZ are dephosphorylated and accumulate in the nucleus, where they bind with TEADs to induce gene transcription. Various upstream mediators, such as adhesion proteins, mechanotransduction, and other signaling pathways, directly or indirectly to regulate the activity of YAP. For relationship among signalings, arrow represents positive regulation and transverse line represents negative regulation. AMOT, angiomotin; GPCR, G protein–coupled receptors; KIBRA, kidney and brain protein; NF2, neurofibromin 2; RASSF1A, ras association domain-containing protein 1A; VGL, vestigial like family.

The Hippo pathway is a kinase cascade that is highly conserved from *Drosophila melanogaster* to mammals. Although there are still many unknowns about the Hippo-YAP pathway, abundant studies have identified that the Hippo pathway primarily functions to regulate cell proliferation and apoptosis to control organ size and tissue homeostasis during animal development and regeneration (26). The core components of the mammalian Hippo pathway include mammalian Ste20-like kinases 1/2 (MST1/2), Salvador (SAV, also called WW45), large tumor suppressor homolog 1/2 (LATS1/2), and the scaffolding proteins MOB domain kinase activator 1A/B (MOB1). MST1/2 and SAV form a complex that can phosphorylate and activate LATS1/2, which then binds to the cofactor MOB1. Then, LATS1/2 further phosphorylates the downstream transcriptional coactivators YAP and TAZ (transcriptional coactivator with a PDZ binding motif). In particular, SAV and MOB1A/B interact with MST1/2 and LATS1/2, respectively, and function as cofactors. YAP can shuttle between the cytoplasm and nucleus, which can be regulated by its phosphorylation status (**Figure 1**). Phosphorylation of YAP Ser127 leads to 14-3-3 binding, cytoplasmic retention (27, 28). And phosphorylation of YAP Ser381 by the Hippo pathway promotes YAP degradation through ubiquitination and reduces its nuclear localization (29). In addition, phosphorylation of YAP at Y357 increases its stability and in turn upregulates its activity (30). In the absence of Hippo pathway inhibition, YAP/TAZ accumulate in the nucleus and interact with transcription factors in which TEA domain transcription factor family members (TEADs) are major partners to regulate the transcription of downstream target genes (26, 31).

### Upstream regulators of the Hippo-YAP pathway

The activity of the Hippo signaling pathway is also regulated by various upstream regulators, such as cell polarity, adhesion proteins, mechanotransduction, and other signaling pathways (the Wnt/β-catenin and GPCR (G protein-coupled receptors) pathways) (**Figure 1**).

Importantly, the Hippo pathway, unlike other classic signal transduction pathways, does not seem to have dedicated receptors and extracellular ligands. Rather, the Hippo pathway is regulated by a network of upstream components that play roles in other processes, such as the establishment of cell–cell adhesion, including adherens junctions and tight junctions (32–37). These cell junctional complexes contain angiomotin (AMOT) family members, which have been shown to interact with the actin cytoskeleton and directly link to the Hippo pathway under different contexts (38–42); E-cadherin and its adaptor protein α-catenin, which interact with the YAP−14-3-3 protein complex (33, 43, 44); and various scaffolding components (such as neurofibromin 2 (NF2; also known as Merlin) and kidney and brain protein (KIBRA; also known as WWC1)), which regulate the activity of the core complex of the Hippo pathway (34, 45–47). Particularly, NF2 directly activate the Hippo pathway during physiological and pathological states in heart (48–50). Thus, disrupting cell–cell adhesion can have strong effects on Hippo pathway activity and lead to the activation or repression of YAP/TAZ (32).

Whether during development or adulthood, tissue homeostasis remains dependent on mechanical cues, and perturbations in extracellular matrix (ECM) stiffness or mutations affecting its perception cause pathological conditions in multiple organs, such as the heart, vessels and the liver (51). The mechanical signals exerted by ECM rigidity and cell shape also regulate the activity of Hippo pathway components (31, 52). Mechanical stress, such as when cells are grown on stiff surfaces or exposed to fluid shear stress, triggers YAP and TAZ nuclear translocation (52, 53). Moreover, integrin complexes act as upstream regulators of the Hippo pathway in response to ECM mechanical stress (54, 55). These findings identify the Hippo pathway as a sensor and mediator of mechanical cues instructed by the cellular microenvironment, but the exact mechanisms are not known and need to be further studied.

Interactions with other signaling pathways are profound regulators of Hippo activity. On the one hand, the Wnt pathway has been shown to be negatively regulated by the Hippo pathway by directly interacting with β-catenin (7, 56). On the other hand, the Hippo pathway acts as a downstream effector of the alternative Wnt signaling pathway (57, 58). In addition, these are GPCRs that are activated by lipids (lysophosphatidic acid and sphingosine-1-phosphate) or hormones (glucagon or adrenaline) and signal through F-actin to regulate YAP/TAZ (59, 60). Thus, YAP and TAZ activation–inactivation is a dynamic process involving multiple signaling pathways.

## The inflammatory response in cardiovascular diseases

The development and prognosis of CVDs, including MI, cardiomyopathy and atherosclerosis, are closely related to immune and inflammatory responses. MI, including myocardial ischemia–reperfusion (I/R), and heart failure (HF), are among the leading causes of death and disability worldwide. MI is accompanied by a finely orchestrated and complex series of inflammatory events, which play critical roles in determining acute MI size and subsequent post-MI adverse LV (left ventricle) remodeling in the heart (61). First, hypoxia during ischemia impairs vascular endothelial cell (EC) integrity and barrier functions, thereby increasing vessel permeability and facilitating the infiltration of leukocytes, including neutrophils, monocytes/macrophages, and lymphocytes (62). Then, the duration of myocardial ischemia induces cellular injury and death to different constituents of the myocardium (cardiomyocytes, endothelial cells and cardiac fibroblasts (CFs)). This in turn initiates an acute proinflammatory response through the concerted action of several processes, including complement cascade activation and damage-associated molecular patterns (DAMPs), which serve as ligands for pattern recognition receptors (PRRs), such as toll-like receptors (TLRs). These factors result in the release of a cascade of proinflammatory mediators (such as cytokines, chemokines and cell adhesion molecules) and the recruitment of inflammatory cells into the ischemic region (63, 64).

Cardiomyopathy can be separated into primary (hypertrophic cardiomyopathy and dilated cardiomyopathy) and secondary (diabetic cardiomyopathy, septic cardiomyopathy) categories, which result in varied phenotypes, including myocardial dysfunction and HF (65). The immune response is closely involved in different cardiomyopathies (66–69). Damage to the myocardium, whether from a genetic or environmental cause, triggers an inflammatory response and recruits immune cells to the heart to repair the myocardium, and these cells can be identified in histopathological samples and myocardial late gadolinium enhancement by cardiac magnetic resonance imaging (69–71). A prolonged and continuous inflammatory response in turn brings about the progression of cardiomyopathy.

Vascular remodeling is a complex process that involves physical, biochemical and genetic components. It is a vital process in a wide range of CVDs, including atherosclerosis, hypertension and diabetes, and often involves the interplay of inflammatory cells (72–75). Under various stimuli, vascular ECs activate proinflammatory signaling and recruit inflammatory cells, especially macrophages, into the vessel, which is accompanied by a series of inflammatory cascade responses (76–78). Thus, targeting inflammation in vascular remodeling might be a promising therapeutic strategy.

## The Hippo-YAP pathway and inflammation during myocardial infarction

**Figure 2:**
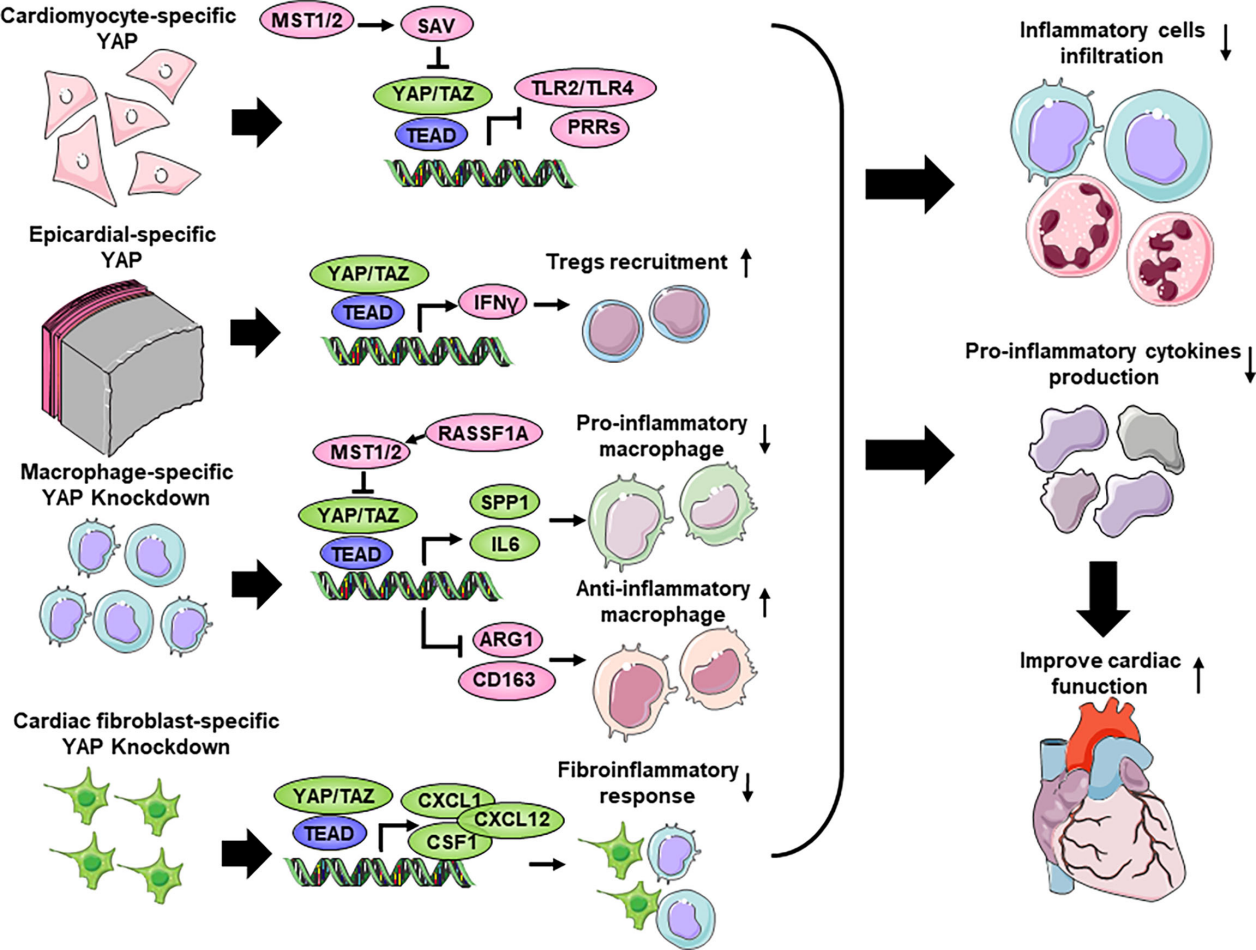
Cell type-dependent Hippo-YAP pathway regulate the inflammation response after Myocardial infarction. Cardiomyocyte-specific YAP inhibits inflammatory cells infiltration, including macrophages and neutrophils, and pro-inflammatory cytokines production through interacting with TLRs (toll-like receptors), thus improve cardiac function. Epicardial-specific YAP increases Tregs (T-regulatory cells) recruitment to inhibit macrophage infiltration and pro-inflammatory cytokines production through affecting IFN-γ (interferon γ) expression. Macrophage-specific knockdown of YAP decreases the accumulation of pro-inflammatory macrophages and enhances the accumulation of anti-inflammatory macrophages through regulating the expression of Spp1 (secreted phosphoprotein 1), IL6 (interleukin 6), ARG1 (arginase 1), CD163 to improve cardiac function. Cardiac fibroblast-specific knockdown of YAP inhibits fibroinflammatory response to decrease the recruitment of inflammatory cells, the production of pro-inflammatory cytokines and improve heart function. CSF1, colony stimulating factor 1; CXCL1, C-X-C Motif Chemokine Ligand 1; CXCL12, C-X-C Motif Chemokine Ligand 12; PRRs, pattern recognition receptors.

Considering the complex context and diverse cell types in the heart after MI, we will discuss the role of the Hippo-YAP pathway in different cell types in the inflammatory response during MI (**Figure 2**).

### Cardiomyocytes

Studies have shown that hypoxia during MI activates Hippo pathway kinases, which increase caspase activation and cardiomyocyte apoptosis and thereby increase the levels of phosphorylated YAP. Cardiomyocyte-specific overexpression of Hippo kinases, such as MST1 (79–81), SAV (6) and LATS2 (82), in mice results in increased cardiomyocyte apoptosis and progressive deterioration of cardiac function, whereas cardiomyocyte-specific overexpression of YAP decreases cardiomyocyte apoptosis and promotes cardiac function (83–85) (**Table 1**). Moreover, in large mammals such as pig, cardiomyocyte-specific knockdown of SAV reduces fibrosis and increases small blood vessels (86). Although multiple studies have depicted the role of the Hippo pathway in regulating cardiomyocyte apoptosis and cardiac function in detail, how Hippo kinases affect inflammatory cell infiltration and inflammation has yet to be defined. Dying cardiomyocytes provide the main stimulus for the postinfarction inflammatory response by releasing DAMPs in the infarcted area (64). Many studies have shown the interaction of the Hippo-YAP pathway and DAMPs in other diseases (98–101); however, to the best of our knowledge, there have been no studies on the relationship between the Hippo-YAP pathway and DAMPs during the progression of MI, which needs to be further studied.

**Table 1:** Hippo-YAP pathway and inflammation in myocardial infarction.

Model	species	Methods	Outcomes	Inflammation outcomes	Ref.
Myocardial infarction	Mouse	Cardiomyocyte-specific overexpression of dominant negative *Mst1*	Improve heart function, decrease infarct size and fibrosis; Decrease CM apoptosis	Decrease proinflammatory cytokines expression; Regulate the transcriptional activity of NF-κB	(79–81)
Myocardial infarction	Mouse	Cardiomyocyte-specific deletion of *Sav*	Improve heart function, decrease infarct size and fibrosis; Increase vascularization	Inflammation is more effectively resolved.	(6)
Ischemia reperfusion	Pig	Knockdown *Sav via* sub-endocardial injection of AAV9-shRNA	Improve heart function, decrease infarct size and fibrosis; Increase capillary density	Without alteration of the number of CD45-positive leukocytes after3-month IR	(86)
Myocardial infarction	Mouse	Cardiomyocyte-specific overexpression of human *Yap* using Cre-flox and AAV9 respectively	Both improve heart function, reduce infarct size; Both increase CM proliferation	In AAV9-targeted mice increase inflammatory marker genes (including Ccl2, Ccl7, Mmp8, and Il1b) at 5days after MI. without alteration of these inflammatory genes at 1 month after MI.	(83)
Myocardial infarction	Mouse	Cardiomyocyte-specific homozygous inactivation of *Yap*	Impair heart function, increase infarct size and fibrosis; Increase CM apoptosis		(84)
Myocardial infarction	Neonatal mouse	Cardiomyocyte-specific deletion of *Yap*	Impair heart function, increase infarct size and fibrosis		(85)
Myocardial infarction	Neonatal mouse	Cardiomyocyte-specific overexpression of YAPS112A	Improve heart function, decrease infarct size and fibrosis; Increase CM proliferation		(85)
Myocardial infarction	Mouse	Cardiomyocyte-specific heterozygous deletion of *Yap*	Impair heart function, increase infarct size; Increase CM apoptosis, decrease CM proliferation		(84)
*In vitro*; LPS	Rat	Activation of human *Yap via* modified mRNA in cardiomyocyte	Reduce CM necrosis	Inhibit expression of a subset of innate immune response genes (TLR2, CD14)	(87)
Ischemia reperfusion	Mouse	Transient activation of human *Yap via* modified mRNA	Improve heart function, reduce scar size; Reduce CM necrosis	Inhibit TLR2 expression; Reduce neutrophils and macrophages infiltration	(87)
LPS	Mouse	Cardiomyocyte-specific knockdown of *Yap* with AAV9	Reduce heart function; without alteration of CM apoptosis	Activate TLR4/NFκB; Without alteration macrophages and neutrophils infiltration	(88)
Myocardial infarction	Mouse	Epicardial-specific deletion of *Yap* and *Taz*	Impair heart function, increase infarct size and fibrosis, increase mortality	Increase pericardial inflammation response; Increase macrophages infiltration; Decrease IFNγ and Treg recruitment	(89)
Ischemia reperfusion	Mouse	Myeloid cell-specific deletion of *Rassf1A*	Impair heart function, increase infarct size and fibrosis	Increase expression of pro-inflammatory cytokines TNFα, IL-1β, Nos2, and Cox2; Increase macrophage infiltration	(90)
Myocardial infarction	Mouse	Myeloid cell-specific deletion of *Yap*/*Taz*	Improve heart function, reduce infarct size and fibrosis; Increase vascularization	Decrease expression of pro-inflammatory gene (IL6) and increase expression of reparative marker gens (Arg1); Decrease pro-inflammatory macrophages infiltration and increase reparative macrophages infiltration; without alteration of neutrophil infiltration	(91)
Myocardial infarction	Mouse	Myeloid cell-specific overexpression of *Yap*	Impair heart function, increase infarct size and fibrosis	Increase expression of pro-inflammatory gene (IL6) and decrease expression of reparative marker gens (Arg1);	(91)
Myocardial infarction	Mouse	Myeloid cell specific deletion of *Mst1/2*	Impair heart function, increase infarct size and fibrosis	Without alteration of macrophages infiltration; Promote macrophage subtype switching and impair inflammation resolution	(92)
Myocardial infarction	Mouse	Fibroblast-specific deletion of *Yap*	Improve heart function, decrease infarct size and fibrosis; Decrease CM apoptosis; Decrease fibroblast activation and proliferation		(93)
Baseline	Mouse	Fibroblast-specific deletion of *Lats1/2*	Impair heart function, spontaneous fibrosis	Sham hearts acquire similar phenotypic expansion of myeloid cells of an injured heart. Increase myeloid cell infiltration and activation and increase pro-inflammatory signaling.	(94)
Myocardial infarction	Mouse	Fibroblast-specific deletion of *Lats1/2*	Impair heart function, increase fibrosis and mortality	Increase pro-inflammatory gene expression	(94)
*In vitro*	Rat	siRNA-mediated knockdown of *Yap* in cardiac fibroblast	Reduce expression of pro-fibrotic genes (SRF, Eln)	Increase expression of inflammation factors (TLR2, IL6)	(95)
Myocardial infarction	Mouse	Fibroblast-specific deletion of *Yap* and *Taz*	Improve heart function, decrease infarct size and fibrosis; Decrease fibroblast activation and proliferation	Decrease pro-inflammatory gene expression including IL33; Decrease monocytes/macrophages infiltration and polarization	(96)
Myocardial infarction	Mouse	Fibroblast-specific overexpression of *Yap*	Impair heart function, increase fibrosis; Increase fibroblast activation		(96)
Baseline	Mouse	Fibroblast-specific overexpression of *Yap via* AAV	Impair heart function, increase fibrosis; Increase fibroblast activation	Increase inflammation markers (CCL2, CCL5, IL1β); Increase macrophages infiltration	(97)

Surviving cardiomyocytes in the infarct border zone may also trigger an inflammatory response by activating PRRs, which mainly include TLRs, by binding to DAMPs to robustly produce and secrete inflammatory cytokines, which further cause the infiltration of inflammatory cells. Importantly, cardiomyocyte YAP/TEAD1 regulate the expression of TLR genes in MI (87, 88, 102). Specifically, cardiomyocyte overexpression of YAP directly suppresses the expression of TLRs, especially TLR2 and TLR4, whereas cardiomyocyte-specific *Yap* depletion increases the expression of TLR2 and TLR4 *in vivo* and *in vitro* (87, 88). Mechanistically, TEAD1 can directly bind genomic regions adjacent to several TLRs, especially the transcriptional start region of *Tlr4* (88). In addition, TLR3-mediated regeneration and repair of the damaged neonatal myocardium occurs through glycolytic-dependent YAP activation (102). Thus, it is worthwhile to further delineate the detailed regulatory mechanisms of the Hippo-YAP pathway and TLR signaling. Cardiomyocyte-specific knockdown of *Mst1* decreases proinflammatory cytokine expression, including TNF-α, IL1, and IL6, and regulates the transcriptional activity of NF-κB in the heart 4 weeks after MI (79). Similarly, using RNA sequencing on hearts with cardiomyocyte-specific knockout of *Sav* and control hearts, the authors showed that the downregulated genes in *Sav*-CKO cardiomyocytes after MI were associated with inflammation, indicating that inflammation was more effectively resolved in *Sav*-CKO mice (6). Chen et al. used modified mRNA (modRNA) to transiently express constitutively active human YAP in the heart, which significantly reduced cardiomyocyte death and myocardial neutrophil and macrophage infiltration after I/R (87). In conclusion, cardiomyocyte YAP decreases proinflammatory cytokine activation and macrophage or neutrophil infiltration by inhibiting TLR signaling.

Recently, a series of studies have demonstrated that the epicardium is activated following myocardial injury (103, 104). The activated adult epicardium is a source of proinflammatory signals after MI (105, 106). T-regulatory cells (Tregs), which are a subset of CD4+ T cells, have been shown to suppress the innate and adaptive immune response and limit deleterious remodeling following myocardial injury (107). Importantly, epicardial deletion of *Yap*/*Taz* exacerbates fibrosis and pericardial inflammation by decreasing the recruitment of suppressive CD4^+^ Tregs and decreasing the expression of genes encoding IFN-γ (interferon γ), a known Treg inducer. However, controlled local delivery of IFN-γ following MI rescued Treg infiltration into the injured myocardium in *Yap*/*Taz* mutants and decreased fibrosis (89). Future studies will be needed to identify inflammatory cytokines that are dysregulated by epicardial YAP/TAZ in the injured myocardium.

### Macrophages

A growing number of studies are emerging about the role of the Hippo-YAP pathway in macrophages in various diseases, including MI (23, 108). Global deletion of *Rassf1A* increases macrophage accumulation and release of the proinflammatory cytokine TNFα in the heart after IR, and this pattern cannot be found in cardiomyocyte-specific *Rassf1A*-knockout mice relative to controls. In addition, myeloid-specific deletion of *Rassf1A* increases the expression of inflammatory cytokines and macrophage infiltration caused by I/R in mice, suggesting that macrophage RASSF1A plays a leading role in regulating cardiac inflammation after I/R. Cell-based studies revealed that RASSF1A negatively regulates YAP to inhibit NF-κB in macrophages and thereby attenuates inflammatory cytokine expression in macrophages (90).

Traditional views of macrophage biology defined macrophages as monocyte-derived cells that are composed of two populations: proinflammatory M1 macrophages and reparative M2 macrophages (109, 110). Early recruitment on Days 1 to 3 after MI of proinflammatory macrophages secreting high levels of proinflammatory cytokines and chemokines such as IL6 contributes to the removal of dead cells. At a later stage on Days 3 to 7 after MI, anti-inflammatory macrophage subpopulations, which are associated with Arg1 (Arginase 1), are selectively recruited and may participate in the resolution of the postinfarction inflammatory response (111). The expression of YAP and TAZ was increased in macrophages undergoing proinflammatory or reparative phenotype changes (91), and the expression of endogenous MST1 in the cardiac macrophages of wild-type mice was decreased in the first 3 days after MI (92), suggesting the potential role of the Hippo pathway in the cardiac inflammatory response after MI. Genetic deletion of *Yap*/*Taz* in macrophages impairs the proinflammatory macrophage phenotype and promotes a reparative macrophage phenotype, which is accompanied by improved post-MI ventricular remodeling and heart function after MI. In contrast, YAP activation in macrophages shows the opposite effects on macrophage polarization and cardiac function. Mechanistically, YAP/TAZ promote the proinflammatory response by directly regulating *Il6* promoter activity and impair the reparative response by binding to the Arg1 promoter (91).

However, macrophages in MI are heterogeneous and are not confined to the M1 and M2 phenotypes (112, 113). Novel techniques that allow the resolution of gene expression at the single-cell level, including single-cell RNA sequencing, improve the understanding of the definitions of macrophage subsets. Combined with single-cell sequencing, Liu et al. reported three macrophage subtypes (MP1, MP2 and MP3) in MI hearts. MP1 cells highly expressed the proinflammatory genes *Ccl4*, *Ccl2*, and *Spp1*. In contrast, MP2 cells had high expression of anti-inflammatory genes such as *Cd163*, *Cbr2*, and *Rgs10*. MP3 cells have mixed properties and express anti-inflammatory (*Slpi* and *Arg1*) and proangiogenic (*Cxcl2*) genes (92). Although no differences were observed in the numbers of infiltrated macrophages, monocytes, or neutrophils in *Mst1/2* myeloid-specific knockout hearts compared to controls, there were differences in the numbers of the three macrophage subtypes, as indicated by increased MP1 cells and decreased MP2 and MP3 cells. In particular, *Mst1* mutant hearts showed increased *Spp1* expression levels and reduced *Cd163* and *Arg1* expression in CD68^+^ cells, indicating that *Mst1/2* knockout promoted macrophage subtype switching and impaired inflammation resolution in the mouse hearts after MI (92). Mechanistically, MST1 phosphorylates 5-lipoxygenase at its T218 residue, resulting in a reduction of leukotriene B4 production. However, in *Mst1-YAP* double knockout macrophage, leukotriene B4 production is similar with *Mst1* knockout control, suggesting macrophage YAP and MST1 might contribute to cardiac repair post-MI independently. In summary, although YAP exhibits an anti-inflammatory phenotype in response to viral infection (21, 114), macrophage YAP exacerbates the inflammatory response and regulates macrophage subtypes in the heart after MI, resulting in impaired cardiac function.

### Cardiac fibroblasts

CFs, which are one of the most abundant cells in the mammalian heart, remain quiescent and may play a role in maintaining the ECM network; however, when stimulated with DAMPs and reactive oxygen species, CFs are capable of secreting large amounts of inflammatory cytokines and chemokines and acquire a proinflammatory phenotype (111). In CFs, the phosphorylation level of YAP is reduced and thus YAP translocates into nucleus post-MI both *in vivo* and *in vitro* (93, 115). TAZ expression is increased in infarct zone after MI, but whether the increase in TAZ expression is originated from activated myofibroblasts still need to be studied (116). Furthermore, Xiao et al. conditionally deleted *Lats1* and *Lats2* (*Lats1/2*) in CFs and used single-cell sequencing to show that *Lats1/2* deletion led to an ongoing CF-derived proinflammatory cascade that promoted myeloid cell influx, activation, and expansion in sham hearts and post-MI hearts (94). Another study showed that siRNA-mediated *Yap* knockdown in primary rat CFs revealed a specific role for Yap in controlling the expression of proinflammatory cytokines and chemokines (*Tlr2*, *Il6*, *C2*, *C4a*) (95). Fibroblast-specific deletion of *Yap*/*Taz* reduces the post-MI inflammatory response and affects the polarization and infiltration of macrophages in the infarcted hearts, further attenuating MI–induced cardiac dysfunction and fibrosis (96). *In vivo*, an adeno-associated virus increased the expression of YAP in CFs, increased chemokine expression (*Ccl2*, *Ccl5*) and promoted macrophage recruitment. *In vitro*, YAP activation in cultured CFs increased profibrotic and proinflammatory gene expression. Further study showed that YAP regulates *Ccl2* expression by binding to the *Ccl2* promoter (97). In summary, YAP in fibroblasts promotes inflammatory reactions and impairs heart function after MI. The differences and mechanism of the Hippo pathway in the inflammatory response and cardiac repair among different cell types after MI need to be clarified in future studies.

## The Hippo-YAP pathway and the inflammatory response during heart failure and cardiomyopathy.

**Figure 3:**
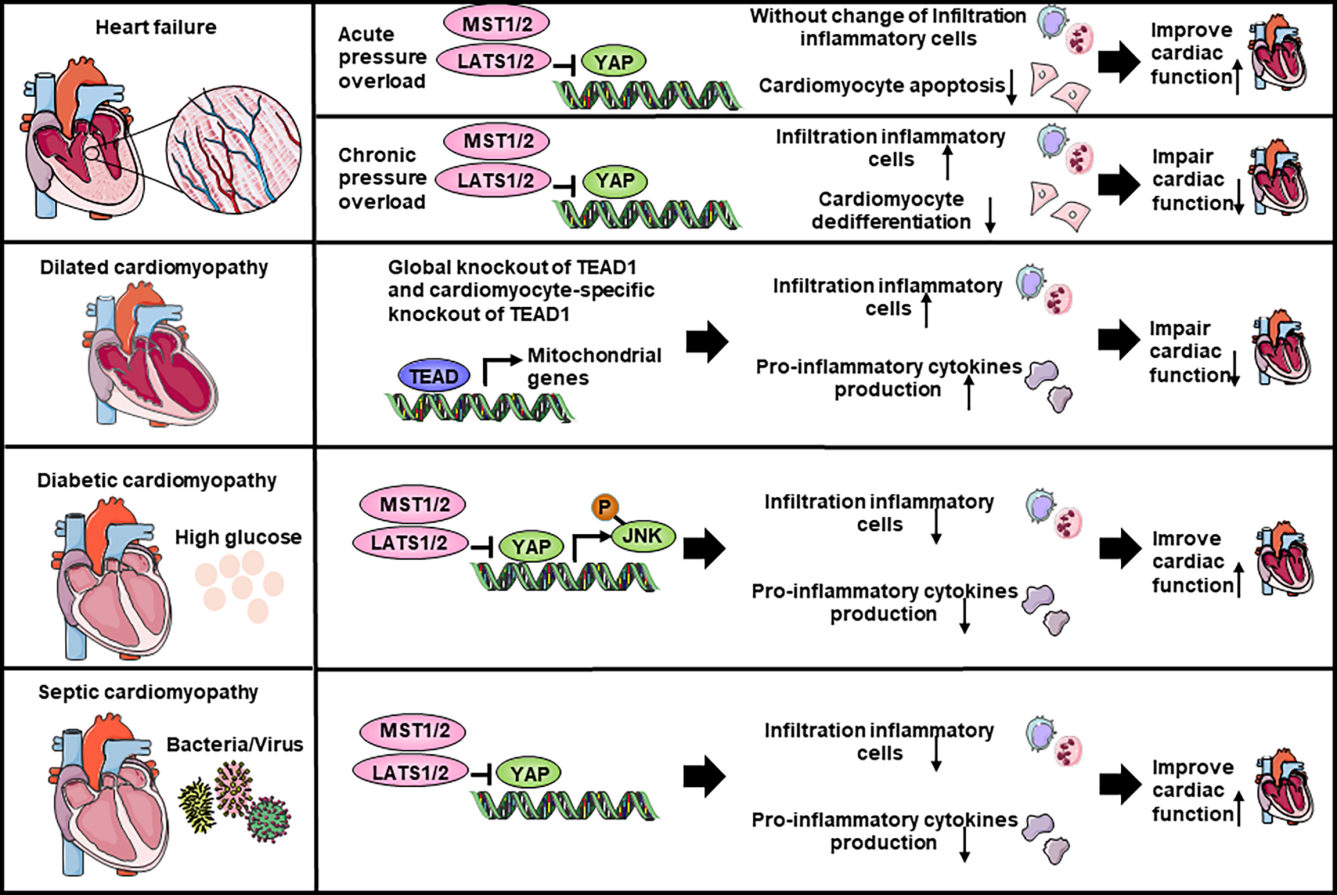
The Hippo-YAP pathway regulate the inflammation response in heart failure and cardiomyopathy. YAP increases the accumulation of inflammatory cells and impairs cardiac function in chronic pressure overload while hardly affects the accumulation of inflammatory cells in acute pressure overload. In dilated cardiomyopathy, global knockout of *Tead1* and cardiomyocyte-specific knockout of *Tead1* both can increase inflammatory cells infiltration and pro-inflammatory cytokines production to impair heart function. Both in diabetic cardiomyopathy and septic cardiomyopathy, YAP is able to inhibit inflammatory cells recruitment and pro-inflammatory cytokines production, thereby improve heart function. JNK, c-Jun N-terminal kinase.

Heart failure affects about 40 million people worldwide (117). Molecular biology experiments showed that YAP is highly expressed and activated in hypertrophic cardiomyopathy tissue samples, as well as in TAC murine hearts, indicating that Hippo/YAP signaling is involved in the pathogenesis of cardiac hypertrophy (118) (**Table 2**; **Figure 3**). Cardiomyocyte-specific genetic modification experiments showed that YAP protected against cardiomyocyte apoptosis and protected heart function during acute pressure overload (PO) but exacerbated the progression of cardiac dysfunction during chronic PO (119–122), suggesting that the effects of YAP differ between the acute and chronic phases of PO. This discrepancy maybe caused by long-term elevation of YAP and concomitant upregulation of TEAD1 which induces cardiomyocyte dedifferentiation. In recent decades, a growing number of studies have suggested that an immune response regulated the development of cardiac hypertrophy (132, 133). Ikeda et al. used cardiomyocyte-specific knockout of *WW45* (*WW45*-cKO) to inhibit the activity of MST1/2 and LATS1/2. Interestingly, myocardial infiltration of leukocytes, macrophages, and neutrophils was not significantly different between control and *WW45*-cKO mice 1 week after PO. The infiltration of macrophages and neutrophils was significantly enhanced 4 weeks after PO in *WW45*-cKO mice compared with control mice (122). In CFs, inhibiting *Rassf1A* promoted fibroblast proliferation, activation of the NF-κB pathway and selective upregulation of TNF-α expression (123), suggesting that the RASSF1A-Hippo pathway affects the inflammatory signaling pathway in fibroblasts. It is vital to further elucidate the mechanism by which the Hippo-YAP pathway regulates immune cell infiltration, including macrophage, Treg, neutrophil, and proinflammatory cytokine activation, after PO.

**Table 2:** Hippo-YAP pathway and inflammation in heart failure and cardiomyopathy.

Model	species	Methods	Outcomes	Inflammation outcomes	Ref.
Baseline	Mouse	Cardiomyocyte-specific overexpression of human *Yap*	Impair heart function enlarge heart, accelerate cardiac hypertrophy		(118)
Transverse aortic constriction	Mouse	Cardiomyocyte-specific heterozygous deletion of *Yap*	Impair heart function, increase CM apoptosis and fibrosis; Decrease cardiac hypertrophy		(119)
Transverse aortic constriction	Mouse	Inhibit MST1/2 *via* inhibitor XMU-MP-1	Improve heart function, increase CM survival, reduce CM apoptosis and fibrosis; Decrease cardiac hypertrophy		(120)
Transverse aortic constriction	Mouse	System heterozygous deletion of *Lats2*	Improve heart function, decrease fibrosis and CM apoptosis; Decrease cardiac hypertrophy		(121)
Transverse aortic constriction	Mouse	Cardiomyocyte-specific deletion of *WW45*	Impair heart function, decrease CM apoptosis; Activate CM cell cycle re-entry	Increase leukocyte, macrophage and neutrophil infiltration 4 weeks after TAC	(122)
Transverse aortic constriction	Mouse	Cardiomyocyte-specific overexpression of *Rassf1A*	Impair heart function, increase fibrosis; Increase CM apoptosis; Without differences in cardiac hypertrophy		(123)
Transverse aortic constriction	Mouse	Cardiomyocyte-specific deletion of *Rassf1A*	Improve heart function, decrease fibrosis; Decrease CM apoptosis; Attenuate cardiac hypertrophic response		(123)
*In vitro*;	Rat	ShRNA mediated knockdown of *Rassf1A*	Promote fibroblast proliferation	Activate NF-κB pathway	(123)
Baseline	Mouse	Global or cardiomyocyte-specific deletion of *Tead1*	lethality due to an acute-onset dilated cardiomyopathy	Increase expression levels of pro-inflammatory cytokines; Increaase expression of macrophage markers	(124)
Diabetic cardiomyopathy	Mouse	MST1/2 inhibitor XMU-MP-1; Global knockout of *Mst1*	Improve heart function		(125–128)
Diabetic cardiomyopathy	Mouse	AAV9 mediated knockdown of *Mst1*	Improve heart function,derease firbosis	Inhibit the levels of inflammatory cytokines which mediated by JNK pathway; Decreases cardiac neutrophil and macrophage infiltration	(129)
Diabetic cardiomyopathy	Mouse	Endothelial-specific overexpression of *Mst1*	Impair heart function and aggravate insulin resistance		(128, 130)
LPS-induced Septic cardiomyopathy	Mouse	Global knockout of *Mst1*	Improve heart function; Decrease CM apoptosis	Attenuate inflammation damage	(131)

Dilated cardiomyopathy is a nonischemic heart muscle disease with structural and functional myocardial abnormalities that can be caused by infection, autoimmunity, endocrine dysfunction, muscular dystrophy, pregnancy and gene mutation (67). Several studies have demonstrated that activation of the Hippo signaling pathway causes dilated cardiomyopathy in mice (85, 134–136). Inflammation occurs during the development of dilated cardiomyopathy, as indicated by the infiltration of inflammatory cells, such as macrophages, and the production of proinflammatory cytokines, which in turn affects the progression of dilated cardiomyopathy. Global knockout or cardiomyocyte-specific knockout of *Tead1* can dramatically increase the expression levels activated macrophage markers and the proinflammatory cytokines *Il6* and *Ccl2* in the heart, which is accompanied by acute onset of dilated cardiomyopathy (124). However, the role of the Hippo-YAP pathway and inflammation in nonmyocytes has not been studied.

Diabetic cardiomyopathy is defined by abnormal myocardial structure and performance in the absence of other cardiac risk factors, such as coronary artery disease, hypertension, and significant valvular disease, in individuals with diabetes mellitus (137, 138). A maladaptive proinflammatory response has been implicated in the development of diabetic cardiomyopathy (137, 139–141). Inhibiting *Mst1* in multiple ways, such as the pharmaceutical inhibitor XMU-MP-1 or transgenic mice, improves glucose tolerance and heart function in a diabetic mouse model (125–129), whereas the overexpression of MST1 impairs cardiac function and exacerbates insulin resistance (128, 130). Specifically, cardiomyocyte-specific *Mst1* inhibition decreases cardiac neutrophil and macrophage infiltration and inhibits the levels of inflammatory cytokines mediated by the JNK (c-Jun N-terminal kinase) pathway during the development of diabetic cardiomyopathy (129). Immunostaining and immunoblot analyses showed that nuclear expression of YAP in cardiomyocytes and the level of YAP protein expression in the heart were significantly higher in HF patients with diabetes than in HF patients without diabetes (142). However, in the diabetic heart, inhibiting YAP significantly attenuated the infiltration of leukocytes, macrophages and neutrophils after PO (142), suggesting that YAP may promote HF by stimulating cardiomyocyte dedifferentiation and inflammation in diabetic hearts in the presence of high blood pressure.

Septic cardiomyopathy, which is characterized by reversible left ventricular systolic dysfunction, is increasingly recognized as a potential complication of septic shock (143). Because sepsis is an inflammatory condition, inflammatory cytokine production is seen to some extent in all affected organs, including the heart (144). LPS (lipopolysaccharide) administration *in vivo* can induce a model of septic cardiomyopathy (145). Global *Mst1* deletion can attenuate LPS-mediated upregulation of TNFα and IL6 levels in the heart, thereby improving cardiac function (131). However, no further study has been performed on the interaction of the Hippo-YAP pathway and septic cardiomyopathy.

## The Hippo-YAP pathway and the inflammatory response during vascular remodeling

Vascular remodeling refers to structural and functional alterations in the arterial wall in response to vascular injury, such as disturbed flow (DF), metabolic disorders, hypertension and exogenous pathogens. Although inflammation is a well-accepted pathological mechanism in vascular remodeling, to date, it has not been translated to specific therapies used in clinical practice (16).

### Vascular endothelial cells

**Figure 4:**
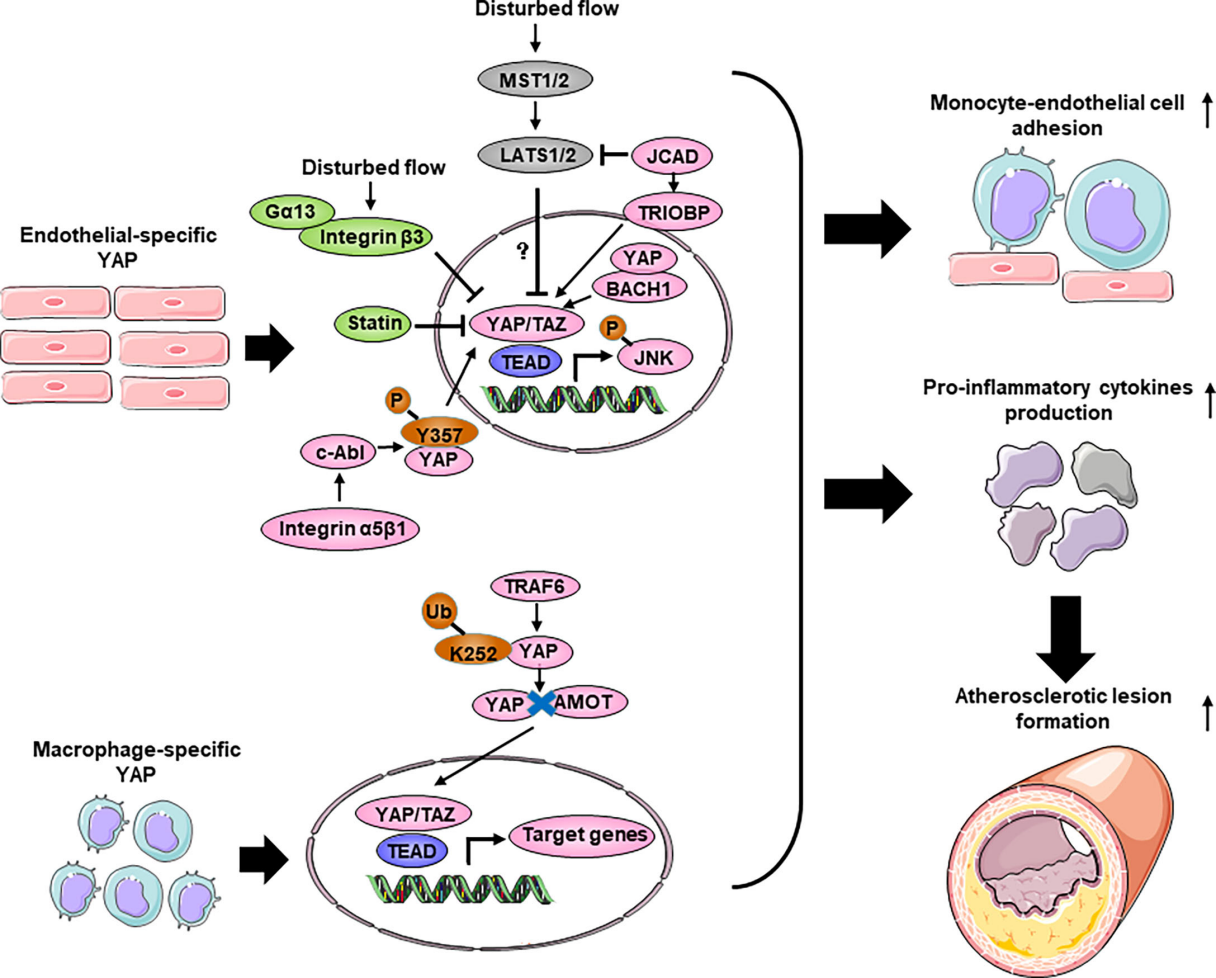
The role of Hippo-YAP pathway regulating inflammation response during atherosclerosis. Disturbed flow promotes YAP nuclear translocation and activates the JNK signaling without affecting the Hippo kinases, thereby accelerates monocyte-endothelial cell adhesion and pro-inflammatory cytokines production to increase the formation of atherosclerotic lesion. Several upstream regulators such as JCAD, BACH1, Integrin β3 and c-Abl affect endothelial inflammation and atherosclerosis through interacting with YAP. Moreover, statins exert anti-atherosclerosis function partly through YAP. In bone marrow derived macrophage, YAP undergoes TRAF6-dependent ubiquitination and translocate into nucleus in response to IL-1β. Macrophage-specific overexpression of YAP promotes macrophage accumulation and pro-inflammatory cytokines production, thereby aggravates atherosclerosis. BACH1, BTB domain and CNC homolog 1; Gα13, guanine nucleotide-binding protein subunit alpha 13; JCAD, junctional cadherin 5 associated; TRIOBP, TRIO and F-actin binding protein; TRAF6, TNF Receptor Associated Factor 6.

Vascular endothelial cells (ECs) covering the inner surface of blood vessels are constantly exposed to shear stress because of the frictional force created by the blood flow (146). Different shear forces induce distinct cellular responses. Regions of arteries with bifurcations, curvatures, or valves have weak shear stress with complex changes in direction, called disturbed flow. The endothelium of these regions have increased permeability, increased turnover and increased monocytes adhesion, thus are susceptible to developing atherosclerotic plaques. In contrast, artery regions of straight segments such as descending thoracic aorta have unidirectional shear stress that is anti-inflammatory and atheroprotective (147). Immunofluorescence staining of the normal carotid artery showed that YAP/TAZ-positive cells were abundantly present in the endothelium in the tunica intima, but few were present in the media layer. In contrast, in the atherosclerotic carotid artery and the aortic arch, YAP/TAZ staining was prominent in both the endothelium, the media layer and the intimal hyperplasia plaque, suggesting that YAP/TAZ may regulate EC functions and that the dysregulation of YAP/TAZ may contribute to the development of atherosclerosis (148) (**Table 3**; **Figure 4**). Several groups showed that pYAP^Ser127^ was increased in HUVECs (Human umbilical vein endothelial cells) and mouse aortas exposed to USS in a time-dependent manner. Accordingly, USS suppressed the transactivation of YAP/TAZ and downregulated the expression of target genes. Conversely, DF reduced pYAP^Ser127^ expression and increased YAP/TAZ target gene expression (54, 148–151). DF promotes inflammation through YAP/TAZ activation (148). YAP/TAZ activation induces several proinflammatory markers, such as *Il6*, *Il8* and *Ccl2* (54, 148, 149, 151), and increases monocyte adhesion to HUVECs, suggesting that endothelial YAP/TAZ activation participates in the initiation of atherosclerosis by promoting monocyte adhesion (54, 148). Furthermore, YAP/TAZ promotes endothelial activation by enhancing JNK activity. EC-specific YAP overexpression in mice significantly increased plaque formation, which was accompanied by increased expression of p-JNK and macrophage markers compared with those of control littermates. Moreover, treatment with the antiatherosclerotic drug statin inhibited EC proliferation and inflammation, which were partly mediated by the inactivation of YAP/TAZ (54, 148).

**Table 3:** Hippo-YAP pathway and inflammation in atherosclerosis.

Model	species	Methods	Outcomes	Inflammation outcomes	Ref.
Disturbed flow	Mouse	Endothelial-specific knockout of *Yap*/*Taz*	Retard progression of atherosclerosis	Decrease monocyte attachment and infiltration; Decrease expression of pro-inflammation cytokines	(54, 148–151)
Disturbed flow	Mouse	Endothelial-specific overexpression of *Yap*/*Taz*	Increase atherosclerotic plaque formation	Promote endothelial inflammation	(148)
High fat diet	Mouse	Endothelial-specific knockout of *Jcad*	Retard progression of atherosclerosis		(152)
High fat diet	Mouse	Mutation the integrin binding site in phosphodiesterase 4D5	Retard progression of atherosclerosis	Decrease monocyte attachment and infiltration; Decrease expression of pro-inflammation cytokines	(153)
High fat diet	Mouse	Endothelial-specific overexpression of *Yap*	Increase atherosclerotic plaque formation	Increase expression of pro-inflammation cytokines	(154)
Disturbed flow	Mouse	Endothelial-specific overexpression of *c-Abl*	Increase atherosclerotic plaque formation	Increase expression of pro-inflammation cytokines	(154)
Disturbed flow	Mouse	Endothelial-specific knockout of *Bach1*	Retard progression of atherosclerosis	Decrease macrophage infiltration; Decrease expression of pro-inflammation cytokines	(155)
High fat diet	Mouse	Endothelial-specific knockout of *Bach1*	Retard progression of atherosclerosis	Decrease macrophage infiltration; Decrease expression of pro-inflammation cytokines	(155)
High fat diet	Mouse	Endothelial-specific knockout of *Jcad*	Retard progression of atherosclerosis	Decrease macrophage infiltration; Decrease expression of pro-inflammation cytokines	(156)
High fat diet	Mouse	Macrophage-specific deletion of *Yap*	Retard progression of atherosclerosis	Decrease macrophage infiltration; Decrease expression of pro-inflammation cytokines	(157)
High fat diet	Mouse	Macrophage-specific overexpression of *Yap*	Aggravate progression of atherosclerosis	Increase macrophage infiltration; Increase expression of pro-inflammation cytokines	(157)

The regulation of YAP/TAZ activities, as indicated by the phosphorylation status of YAP and its cytoplasmic/nuclear localization, are controlled canonically by Hippo pathway kinases, such as LATS1/2 (5). To determine whether flow-induced YAP translocation is dependent on Hippo kinase, researchers used siRNA to knockdown *Lats1*. Interestingly, *Lats1* knockout was not sufficient to reduce laminar shear stress-induced YAP phosphorylation in ECs (148, 150). Recent reports have shown that mechanical stimuli modulate YAP/TAZ activities *via* signaling pathways independent of the Hippo pathway. However, Xu et al. found that USS-mediated YAP^Ser127^ phosphorylation was almost abolished by knockdown of *Lats1/2* expression in HUVECs (149), indicating a critical role of LATS1/2 in mediating laminar flow-induced YAP phosphorylation. The differences among these studies may be attributed to the stimulation conditions, cell states and reagents. Recently, Jones et al. showed that JCAD (Junctional Cadherin 5 Associated) regulates adhesion molecule expression and monocyte-EC adhesion by interacting with LATS2, which is a kinase in the Hippo pathway, in a LATS2-dependent manner (152). However, the administration of an inhibitor of the Hippo kinase MST1/2 resulted in the attenuation of Angiotensin II-induced ascending arterial aneurysms without affecting aortic atherosclerotic plaque progression (158). Due to mouse models and the effects of exogenous inhibition, further direct investigation of the role of Hippo kinase in atherosclerosis is needed. Thus, further investigation will be required to clarify the kinases regulation of the Hippo-YAP pathway in ECs in response to different flow patterns.

In recent years, several upstream molecules have been shown to interact with YAP to regulate atherosclerotic plaque development and inflammation in ECs. The integrin family of receptors plays a critical role in cellular crosstalk with the microenvironment (159). Wang et al. demonstrated that the overexpression of integrin β3 by cytoplasmic-domain-deleted integrin (β3Δcyto) reversed DF-induced YAP phosphorylation in HUVECs, and knockdown of integrin β3 or the G-protein subunit Gα13 attenuated YAP phosphorylation and upregulated YAP/TAZ target gene expression (54). Other groups demonstrated that integrin α5β1 regulated YAP activity in response to DF (153, 154). Li et al. showed that the activation of integrin α5β1 induced tyrosine but not serine phosphorylation of YAP in ECs. Blocking integrin α5β1 with ATN161 abolished the DF-induced phosphorylation of YAP at Y^357^. Mechanistic studies showed that a c-Abl inhibitor attenuated integrin α5β1–induced YAP tyrosine phosphorylation (154), revealing that the integrin α5β1/c-Abl/YAP pathway may be a potential therapeutic target for early-stage atherosclerosis. BACH1 is induced and translocates into the nucleus in HUVECs and upregulates YAP expression by binding to the YAP promoter to form a complex that induces the adhesion of monocytes/macrophages (155). JCAD upregulates the activation of the YAP/TAZ pathway and the expression of downstream proatherogenic genes by interacting with the actin-binding protein TRIOBP (156). The main antiatherosclerotic drugs are statins, which are cholesterol-lowering compounds and are commonly used as first-line treatments for patients with CVDs. Intriguingly, the anti-inflammatory and anti-plaque effects of statins are now being reinterpreted as being mediated, at least in part, by their capacity to inhibit YAP and TAZ (54, 148, 160). More broadly, this finding suggests that endothelial YAP/TAZ could be specifically targeted to treat atherosclerosis.

In conclusion, YAP promotes endothelial inflammation under shear stress-induced atherosclerosis. However, the role of Hippo-YAP in endothelial inflammation is stress dependent. Diabetes-induced endothelial dysfunction and inflammation are critical factors in the mof diabetic vascular complications (161). YAP is dephosphorylated/activated by high glucose in ECs, leading to increased endothelial inflammation and monocyte attachment. Consistently, inhibiting YAP in HUVECs reduces endothelial inflammation and monocyte attachment (162, 163). Knocking down *Yap*/*Taz* in ECs using siRNA significantly reduced the TNF-α-induced expression of the leukocyte adhesion molecule VCAM1 (vascular cell adhesion protein 1) (163). Moreover, YAP aggravates the inflammatory response in the vascular endothelium under high glucose conditions. Similarly, in the aortas of Angiotensin II -induced hypertensive mice, treatment with the YAP/TAZ inhibitor verteporfin reduces macrophage infiltration and proinflammatory cytokine production (164). However, another study demonstrated that Tie2-mediated endothelial-specific knockout of *Yap* in mice resulted in mild endothelial inflammation and impaired microvessel permeability in lung, as evidenced by increased numbers of total cells, neutrophils, and macrophages and an increased neutrophil/macrophage ratio after LPS administration. Mechanistically, in lung ECs, exposure to LPS induces endothelial activation through TLR4 and triggers proinflammatory cytokine secretion and the expression of adhesion molecules *via* NF-κB signaling (165). The reasons for the differences among different groups are not fully clear. It is possible that the differences are because of the cell types and the inflammatory stimuli used.

### Macrophages

During the progression of atherosclerosis, circulating monocytes enter sites of arterial hemodynamic stress by adhering to the ECs lining the lumen of susceptible arteries. Once monocytes enter the subendothelial space, they differentiate into lesional macrophages and further transform into foam cells (166). *Yap*/*Taz* knockdown inhibited the upregulation of β2 integrin and ICAM1 (intercellular adhesion molecule-1) induced by TNFα in THP1 cells and reduced their adhesion to the activated ECs without altering the plasticity of THP1 monocyte-to-macrophage differentiation (148). YAP undergoes TRAF6-dependent Lys63 chain ubiquitination in response to IL-1β in bone marrow-derived macrophages (BMDMs), which results in increased YAP nuclear localization and protein stability. *In vivo*, YAP overexpression in monocytes/macrophages promotes atherosclerotic lesion size and macrophage infiltration, whereas *Yap* deletion in monocytes/macrophages alleviates atherosclerotic plaque development. YAP significantly upregulates the production of chemokines, such as *Ccl2*, *Ccl7*, *Cxcl1*, *Cxcl3*, *Cxcl5* and *Cxcl12*, and monocyte/macrophage migration (157).

## Conclusions

Many studies have revealed the central regulatory role of the Hippo-YAP pathway in various cardiovascular physiological and pathological contexts. Hippo signaling has been shown to exert various effects according to different cell contexts and microenvironments because of its diverse interactions with a variety of signaling transduction cascades. Recently, many interesting findings have suggested that the Hippo-YAP pathway regulates inflammatory cell infiltration and inflammatory cytokine activation, which provides a deeper understanding of the Hippo-YAP pathway in the complex multicellular interaction environment. However, several issues need to be addressed in future studies. First, in cardiovascular research, most of the available studies focused on the role of Hippo-YAP in monocytes/macrophages, and little research has been done on other immune cells, such as T cells, which have been observed in other diseases (18, 167–169). Second, uncovering the classical and nonclassical regulation of the Hippo-YAP pathway in the inflammatory response is essential for controlling CVD progression. Third, future work must bring forth the data to further understand the mechanisms involved and address the cell-specific differences in the role of the Hippo-YAP pathway in CVDs. In summary, an increasing number of uncovered mechanisms of the Hippo-YAP pathway have broadened our horizon, leading to deeper thinking and the search for clinical research transitions.

## Author contributions

QC and LZ conceived ideas. AZ created the figures and wrote the article. All authors contributed to the article and approved the submitted version.

## Funding

This work was supported by a grant from the National Natural Science Foundation of China (81930010, 82125005).

## Acknowledgments

We acknowledge the use of Servier Medical Art image bank that is used to create schematic **Figures 1**–**4**.

## Conflict of interest

The authors declare that the research was conducted in the absence of any commercial or financial relationships that could be construed as a potential conflict of interest.

## Publisher’s note

All claims expressed in this article are solely those of the authors and do not necessarily represent those of their affiliated organizations, or those of the publisher, the editors and the reviewers. Any product that may be evaluated in this article, or claim that may be made by its manufacturer, is not guaranteed or endorsed by the publisher.

## Abbreviations

AMOT, angiomotin; ARG1, arginase 1; BACH1, BTB domain and CNC homolog 1; CBR2, carbonyl reductase 2; CCL, C-C Motif Chemokine Ligand; CF, cardiac fibroblast; CM, cardiomyocyte; CVDs, cardiovascular diseases; CXCL, C-X-C Motif Chemokine Ligand; DAMPs damage-associated molecular patterns; DF, disturbed flow; EC, endothelial cell; ECM, extracellular matrix; GPCR, G-protein–coupled receptors; Gα13, guanine nucleotide-binding protein subunit alpha 13; HF, heart failure; HUVECs, human umbilical vein endothelial cells; ICAM1, intercellular adhesion molecule-1; IFN-γ, interferon γ; IL, interleukin; I/R, myocardial ischemia–reperfusion; JCAD, junctional cadherin 5 associated; JNK, c-Jun N-terminal kinase; KIBRA, kidney and brain protein; LATS1/2, large tumor suppressor homolog 1/2; LPS, lipopolysaccharide; LV, left ventricle; MI, myocardial infarction; MOB1, MOB domain kinase activator 1A/B; MST1/2, mammalian Ste20-like kinases 1/2; NF2, neurofibromin 2; NF-κB, nuclear factor-κB; PRRs, pattern recognition receptors; PO, pressure overload; RASSF1A, ras association domain-containing protein 1A; RGS10, regulator of G protein signaling 10; SAV, salvador; siRNA, short interfering RNA; SLPI, secretory leukocyte peptidase inhibitor; SPP1, secreted phosphoprotein 1; TAZ, transcriptional coactivator with a PDZ binding motif; TEADs, TEA domain transcription factor family members; TLRs, toll-like receptors; TNFα, tumor necrosis factor α; TRAF6, TNF receptor associated factor 6; Tregs, T-regulatory cells; TRIOBP, TRIO and F-actin binding protein; USS, unidirectional shear stress; VCAM1, vascular cell adhesion protein 1; WW45-cKO, cardiomyocyte-specific knockout of WW45; YAP, yes-associated protein.

## Glossary

**Table:**

AMOT	angiomotin
ARG1	arginase 1
BACH1	BTB domain and CNC homolog 1
CBR2	carbonyl reductase 2
CCL	C-C Motif Chemokine Ligand
CF	cardiac fibroblast
CM	cardiomyocyte
CVDs	cardiovascular diseases
CXCL	C-X-C Motif Chemokine Ligand
DAMPs	damage-associated molecular patterns
DF	disturbed flow
EC	endothelial cell
ECM	extracellular matrix
GPCR	G-protein–coupled receptors
Ga13	guanine nucleotide-binding protein subunit alpha 13
HF	heart failure
HUVECs	human umbilical vein endothelial cells
ICAM1	intercellular adhesion molecule-1
IFN-g	interferon g
IL	interleukin
I/R	Myocardial ischemia–reperfusion
JCAD	junctional cadherin 5 associated
JNK	c-Jun Nterminal kinase
KIBRA	kidney and brain protein
LATS1/2	large tumor suppressor homolog 1/2
LPS	lipopolysaccharide
LV	left ventricle
MI	myocardial infarction
MOB1	MOB domain kinase activator 1A/B
MST1/2	mammalian Ste20-like kinases 1/2
NF2	neurofibromin 2
NF-kB	nuclear factor-kB
PRRs	pattern recognition receptors
PO	pressure overload
RASSF1A	ras association domain-containing protein 1A
RGS10	Regulator of G protein signaling 10
SAV	salvador
siRNA	short interfering RNA
SLPI	secretory leukocyte peptidase inhibitor
SPP1	secreted phosphoprotein 1
TAZ	transcriptional coactivator with a PDZ binding motif
TEADs	TEA domain transcription factor family members
TLRs	toll-like receptors
TNFa	tumor necrosis factor a
TRAF6	TNF receptor associated factor 6
Tregs	Tregulatory cells
TRIOBP	TRIO and F-actin binding protein
USS	unidirectional shear stress
VCAM1	vascular cell adhesion protein 1
WW45-cKO	cardiomyocyte-specific knockout of WW45
YAP	yesassociated protein
